# Early prediction of hemodialysis complications employing ensemble techniques

**DOI:** 10.1186/s12938-022-01044-0

**Published:** 2022-10-11

**Authors:** Mai Othman, Ahmed Mustafa Elbasha, Yasmine Salah Naga, Nancy Diaa Moussa

**Affiliations:** 1 Biomedical Engineering Department, Medical Research Institute, Alexandria University, 165, Horreya Avenue, Hadara, Alexandria Governorate, Alexandria, Egypt; 2 Internal Medicine Department, Faculty of Medicine, Alexandria University, Champollion Street, El-Khartoum Square, El Azareeta Medical Campus, Alexandria Governorate, Alexandria, Egypt

**Keywords:** Hemodialysis complications, Machine learning, Feature selection, Ensemble

## Abstract

**Background and objectives:**

Hemodialysis complications remain a critical threat among dialysis patients. They result in sudden termination of the session which impacts the efficiency of dialysis. As intra-dialytic complications are the result of the interplay of multiple factors, artificial intelligence can aid in their early prediction. This research aims to compare different machine learning tools for the early prediction of the most frequent hemodialysis complications with high performance, using the fewest predictors for easier practical implementation.

**Methods:**

Fifty different variables were recorded during 6000 hemodialysis sessions performed in a regional dialysis unit in Egypt. The filter technique was used to extract the most relevant features. Then, five individual classifiers and three ensemble approaches were implemented to predict the occurrence of intra-dialytic complications. Different subsets of 25, 12 and 6 from the 50 collected features were tested.

**Results:**

Random forest yielded the highest accuracy of 98% with the least training time using 12 features in a balanced dataset, while the gradient boosting allowed obtaining the highest F1-score of 94%, 92%, and 78% in the prediction of hypotension, hypertension, and dyspnea, respectively, in imbalanced datasets.

**Conclusion:**

Applying different machine learning algorithms to big datasets can improve accuracy, reduce training time and model complexity allowing simple implementation in clinical practice. Our models can help nephrologists predict and possibly prevent dialysis complications.

**Supplementary Information:**

The online version contains supplementary material available at 10.1186/s12938-022-01044-0.

## Introduction

End-stage kidney disease (ESKD) is a common global problem. In 2019, more than five million people needed renal replacement therapy (RRT) worldwide [1]. Although dialysis is the most utilized RRT modality, it does not replace all the functions of the kidney, leading to multiple long-term complications [2]. In addition to the long-term complications, patients often experience complications during dialysis, which may lead to early termination of the dialysis session decreasing the adequacy of dialysis and affecting quality of patient care. Accordingly predicting such events may allow closer monitoring and timely intervention to avoid these complications [3].

Patients on maintenance hemodialysis attend dialysis centers three times per week. Routinely, the patient is assessed before every session, and laboratory investigations are performed periodically. These data are readily available and can be digitally recorded. However, there is no uniform way to interpret the data and to use it in identifying patients at increased risk for complications [4]. Although statistics may be used to identify individual factors increasing the risk of complications, they cannot accurately predict future events using the multiple interconnected variables.

Machine learning (ML) is a subset of artificial intelligence. It is a complex automatic process of discovering meaningful information from raw data and then turning it into knowledge [5]. ML allows computers to accurately predict outcomes without being explicitly programmed to do so. With the rise in data recording in electronic medical records, there is a growing need for use of machine learning to analyze the big datasets to improve care in various aspects of medicine [6, 7].

In a previous paper [8], our team developed an artificial neural network model (which is also known as multi-layer perceptron (MLP)) to predict the occurrence of 7 intra-dialytic clinical events (see Additional file 1: Table S2 for target outcomes) utilizing a test set collected from a regional tertiary dialysis unit in Alexandria, Egypt. The objective was to create a multiclass prediction model that can distinguish between 8 different classes efficiently (no complications or one of the seven studied complications). So, MLP was chosen because of its efficiency in the multiclass discrimination. The model resulted in 82% accuracy in the multiclass scenario and 96% accuracy in the binary classes scenario using 26 of the 50 recorded. However, recording 26 features for its patient would be a burden on medical staff.

The objective of this paper was to examine whether feature selection, other individual classifiers and ensemble models would perform better than the previously used ANN model to ultimately develop a more accurate model with the fewest features possible and the least complexity.

The rest of the paper is organized as follows: “Related work” section; “Materials and methods” section; “Results” section; “Discussion” section; and finally, “Conclusion” section.

## Related work

Nowadays, data collection is not an issue. The true challenge lies in converting this raw data into useful information. The use of ML techniques assists in the automatic extraction of hidden data patterns that are obscured in the collected data, producing useful knowledge that can affect decision-making. Classification is the most widely used machine learning technique. Machine learning is increasingly utilized in various fields, including healthcare [5], sentimental analysis [7], finance [9], agriculture [10], and social science [6]. But various machine learning techniques exist, each with different advantages and disadvantages and the choice of which is better for each dataset is poorly defined.

Classification allows the grouping of new observations to categories based on a training dataset labeled by category membership. In our case, the classification problem consists of identifying which patients will experience complications during the dialysis session. There are several classification algorithms such as support vector classifier (SVC) [11, 12], Multi-layer Perceptron (MLP) [13, 14], K-Nearest Neighbor (KNN) [7] and Decision Tree (DT) [10, 13]. Furthermore, ensemble of classifiers could be applied [15–20].

The SVC method seeks a hyperplane that optimally divides two classes by taking into account the "sum of the distances from the hyperplane to the nearest positive and negative correctly identified samples" [11]. If the hyperplane can be found in the original data rather than higher-dimensional space, an SVC with a linear kernel is implemented (SVCL) [12]. In nonlinear scenarios, the usage of several kernel approaches aids in dealing with computational complexity difficulties.

MLP network (also called Artificial Neural Network ANN) consists of an input layer, one or more hidden layers, and an output layer [13, 14]. Each node, or artificial neuron, is connected to others and has a weight and threshold that go along with it [8]. Any node whose output exceeds the defined threshold value is activated and begins providing data to the next layer. Otherwise, no data is sent to the network's next tier. Neural networks through training data learn and improve their accuracy over time. However, the training of MLP is the most complex one and time consuming.

KNN [7], the nearest neighbor classification strategy classifies the data based on the type of the closest neighbor. In KNN, the K represents the closest neighbors of the record (to be classified), where classification is carried on the majority class of (K) neighbors.

DT is a tree-like structure [13, 15], in which the data classification process is based on the feature values. The nodes and branches of the tree represent features and output of tested rule, respectively. The attribute of splitting optimizes the selected criteria. It is a two-stage classifier: the first step is concerned with tree construction, while the second stage is concerned with data categorization.

Ensemble techniques are methods that enhance the prediction accuracy by combining multiple models instead of using a single model [18]. The ensemble methods differ in the way of combining models and include voting [17], bagging [15, 19] and boosting [16, 20] approaches.

An ensemble is a machine learning model that integrates the output decision from two or more learners (classifiers). Each classifier may train on different samples of training dataset, with different features and may be with different algorithm. By combining multiple learners and taking full advantage of these learners, ensemble algorithms can enhance results and reduce the overfitting issue.

Ensembles are used to achieve better predictive performance on a classification problem than a single predictive model. Practical and theoretical evidence in previous studies [15–20] show that ensemble techniques can balance the bias–variance trade-off in a model and produces a model with appropriate complexity and flexibility. The ultimate goal would be to create a model with low bias and low variance, which is very challenging in practice. Simplifying the assumption used in a model would lead to more bias, where bias represents the amount that a model’s prediction differs from the actual outcome. The variance of the model is the amount the performance of the model changes when it is fit on different training data. It captures the influence of data on the model. Variance may cause overfitting, in which small variations in the training data are magnified. The diversity of models present in the ensemble concept reduces the bias and variance errors of the ML model and makes the final model more generalizable to be applied to any future unseen dataset.

In dialysis patients specifically, several studies examined the utilization of the mentioned classification techniques in different aspects of patient care. Mezzatesta et al. [5] compared between logistic regression (LR), k-nearest neighbor (KNN), decision tree (DT), naïve Bayes (NB), support vector classifier with linear (SVCL), radial basis function (SVCR), and polynomial (SVCP) kernels for predicting the incidence of cardiovascular diseases in patients on dialysis. The highest accuracy was achieved by SVCR at a level of 95.25%. Yang et al. [21] tried to predict the risk of mortality in hemodialysis patients. In their study, support vector machine (SVM) yielded better accuracy compared to KNN, LR, a linear discriminant, DT, and ensemble. Another study by Xiong et al. [22] proposed seven classifiers and three ensemble learners for predicting the time of initiation of hemodialysis in ESKD. The ensemble learning model they created had the best accuracy which was 97.04%.

Putra et al. [23] constructed MLP model using 3237 sessions. They measured three vital predictors by a special device that is not widely available beside patient demographics. The five events recorded during dialysis were combined into a binary classification scenario (event vs. nonevent). Their model had a mean precision and recall of 93.45%. However, they did not try to predict any individual event and a special device is needed to collect the needed measurement for the model.

Thakur et al. [24] used a special sensor device to monitor vital parameters like the heart rate, heart rate variability, and respiration rate variability of studied patients, then compared between multiple single and ensemble classifiers to predict event or no-event based on the sensor data and demographic information. The Adaptive Boosting ensemble model had the highest performance in all the studied scenarios. Yet, this study required the use of devices and sensors that are not widely available in dialysis units limiting their wide application, unlike in our study which utilizes easily measured variables. Furthermore, only 3 vital features were used for prediction.

In a study by Titapiccolo et al. [25], a logistic regression (LR) model and a random forest (RF) model were applied for predicting the cardiovascular outcome in hemodialysis patients. Besides vital signs, data related to the dialysis machine were used for building the ML models. RF, an ensemble technique, showed higher performance than logistic regression with a sensitivity of over 70%, which relatively still low. Lee et al. [26] created a model capable of real-time prediction of intra-dialytic hypotension using multiple features including repeated vitals. They applied a deep learning model using data from 261,647 hemodialysis sessions. Their ANN model achieved an AUC of 0.94.

To predict acute kidney injury (AKI) after heart surgery, Lee et al. [27] successfully used 6 techniques of classifiers, including SVM, RF, DT, gradient boosting (GB), Artificial Neural Network (ANN), and deep learning, where GB showed the best performance with the highest AUC. On the other hand, Zubair et al. [28] compared the results of different ensemble techniques such as adaptive boosting (AdaBoost), bagging, extra trees, GB, and RF classifiers in detecting chronic kidney patients. The accuracy of AdaBoost, which was 99%, exceeded all other machine learning algorithms.

The mentioned studies utilized diverse techniques to analyze the available data and these different techniques gave different results. Yet most studies were conducted retrospectively and on limited or defective datasets. Our study aims to re-examine our dataset utilizing ensemble techniques, which in most of the previous works resulted the highest performance, to compare them to other individual classifiers and to identify a more simple and accurate model with the fewest features possible.

## Materials and methods

**Fig. 1:**
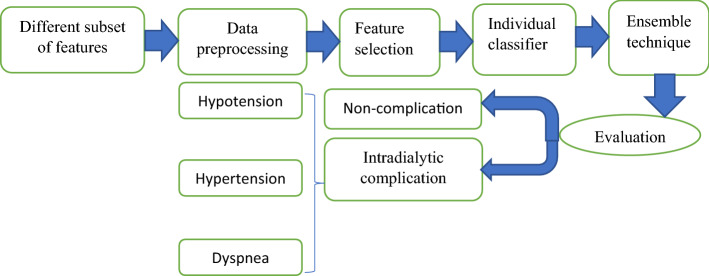
The procedure of the study

The steps followed in our study are illustrated in Fig. 1. All experiments were coded in Python 3.6 using Jupyter notebook IDE and the scikit-learn library. The laptop used had a 2 gigahertz Intel core i7 and 8 gigabytes of RAM, running on Windows 10 operating system.

### Dataset

A total of 215 adult patients between 18 and 60 years of age on maintenance hemodialysis at the dialysis unit of El-Mowasah University Hospital, Alexandria, Egypt, were recruited for the study. Fifty features were collected during 6000 HD sessions performed on the patients between November 2019 and May 2020. Each dialysis session lasted between 3 to 5 h. Features recorded included: features related to the patient’s clinical condition, machine settings, as well as novel features such as room temperature; humidity; and meal, fluid, and caffeine intake during the session (see Additional file 1: Tables S1 and S2).

Four binary datasets were extracted from the main data according to the predicted outcome. Each dataset was randomly divided into 80% training subset (4800 samples) and 20% testing subset (1200 samples) for internal validation of the developed models. The studied datasets are as follows:

- *The first dataset* is divided into two binary balanced groups based on the occurrence of complications. All the seven complications were combined to form a positive class (47.9% of sessions, 2874 sessions) as represented in Additional file 1: Table S2, while in 52.1% of sessions (3126 sessions), patients did not experience any complication.
- *The second dataset* is divided into two binary imbalanced groups based on the occurrence of the most frequent complication, intra-dialytic hypotension (defined as a decrease in systolic blood pressure ≥ 20 mmHg with the presence of symptoms like abdominal pain, vomiting, or restlessness [5]). Hypotension occurred in 16.4% of the sessions, while it did not occur in 83.6%.
- *The third dataset* is divided into two binary imbalanced groups based on the occurrence of intra-dialytic hypertension (defined as an increase in mean arterial pressure of at least 15 mmHg within or after dialysis [6]). Hypertension occurred in 7.4% of the sessions, while it did not occur in 92.6%.
- *The fourth dataset* is divided into two imbalanced groups based on the occurrence of dyspnea, shortness of breath, during the session. Dyspnea occurred in 3.3% of the sessions, while it did not occur in 96.7%.

### Dataset preprocessing

Data preprocessing is a method of converting the raw data into a suitable form for ML to enhance the learning ability of a classifier. First, categorical features were checked and converted into numerical form. Then, the missing data were replaced by using standard statistical methods. Finally, normalizing quantitative data is necessary before the learning process, as different ranges of values would lead to the domination of the higher features over the smaller ones. The MinMax scaler normalization was chosen [13], where all values were converted to a scale between 0 and 1.

### Feature selection

Feature selection is selecting relevant and non-redundant features to improve performance and reduce the overfitting of noisy data. In the real world, choosing the most significant features decreases the complexity of the model exponentially, and enhances data visualization. In medical diagnosis, it is crucial to detect the most critical risk factors related to the disease. We chose to use the filter technique, which is the most frequently used feature selection technique for clinical data [29]. The filter method picks up the relevant features based on the statistical process. Consequently, there is no dependency between the prediction classifier and the selected subset [30]. It is based on ranking the features according to their usefulness in the prediction and evaluating the importance of features based on the properties of data. Then, the output of the selected features is applied to the machine learning algorithm [31]. The filter technique is fast, efficient, and preferred in the voluminous dataset [32].

### Machine learning models

**Fig. 2:**
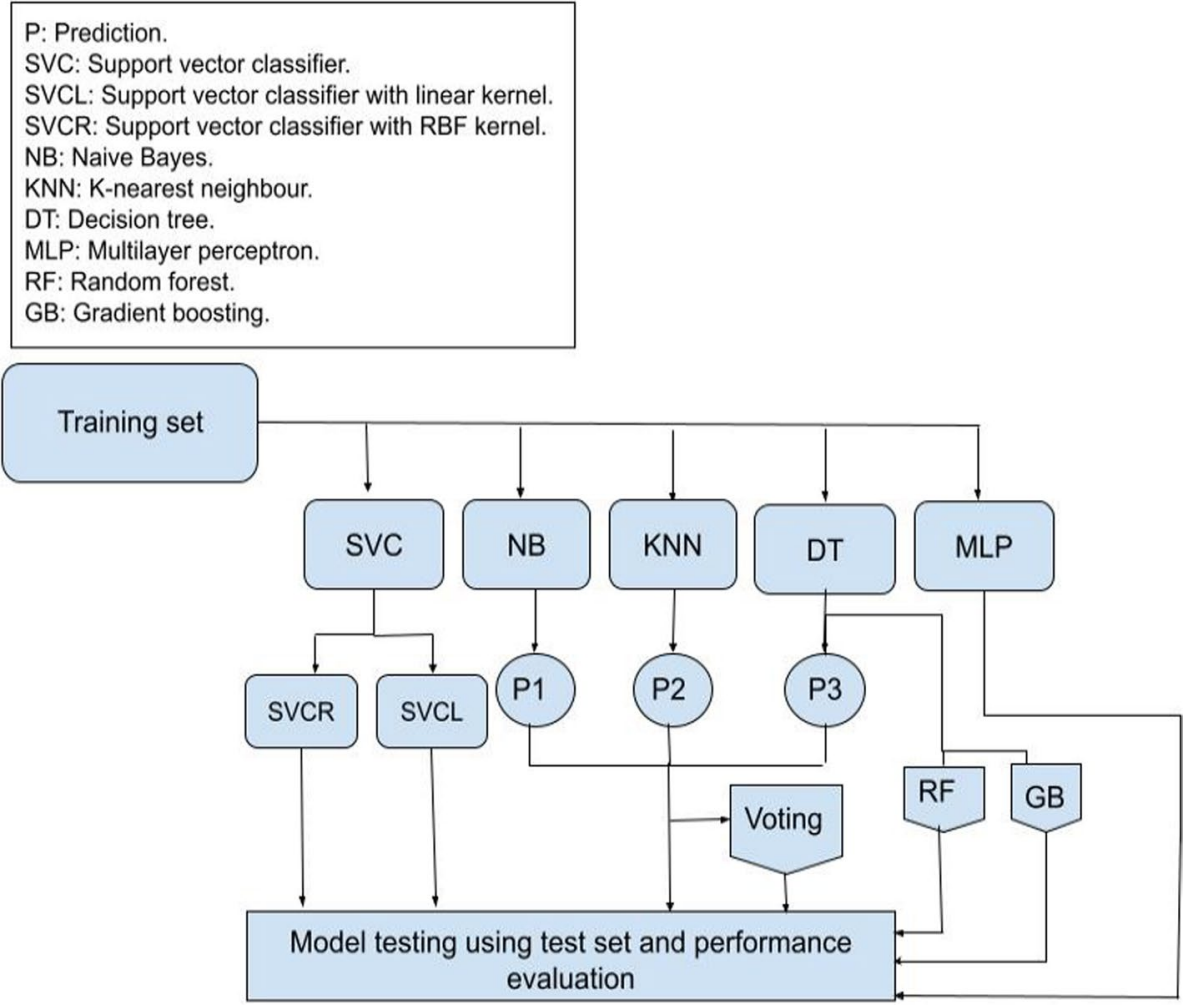
The flowchart of the applied machine learning models

The objective of this research is to compare the performance of different 8 classification methods in developing a model with the best accuracy, accepted complexity and the fewest predictors, so that it can be simply applied in hemodialysis units. We used five individual machine learning models, namely SVCL [11]; SVCR with RBF kernel [12]; MLP (ANN) [14] with 128 neurons, sigmoid function as an activation layer, and binary cross-entropy as a loss function; KNN with *k* = 3; and DT [15] with Gini impurity to measure the incorrect samples. Figure 2 shows the flowchart of the applied machine learning models.

In this work, three ensemble approaches were applied based on bagging [15], boosting [16], and majority voting [17] as summarized in Fig. 2. Majority voting combines the prediction outcomes of different individual classifiers$,$ then selects the outcome that has the most votes. Bagging trains the base classifiers each on random samples extracted from the training set with replacement [15], then aggregates their outcomes by voting to produce the final output. This is the main difference between bagging and majority voting which uses the whole training set with each classifier [18]. Boosting is the combination of weak classifiers to reduce the error [19]. The main theory of boosting is focusing on training samples that are hard to classify. The weak learners learn from misclassified training samples to improve the performance of the ensemble. It is a sequential model, where the first phase is learned from the whole training set. The following phases are learned on the set which is produced based on the performance of the previous one [20]. Boosting employs a variety of loss functions. Adaptive Boosting [16] (referred here in this paper results as Boosting) tries to reduce the exponential loss function which might render the algorithm susceptible to outliers. However, Gradient Boosting (GB) [17], another boosting approach, uses differentiable loss function. Gradient Boosting applies gradient descent optimization for the loss function and is more resistant to outliers.

The difference between bagging and boosting is that the base learner in bagging is standalone; it doesn’t depend on the errors of previous models [20]. Random Forest [15] is considered as a Bagging classifier. It is a particular type of ensemble classifier that exclusively uses decision trees. The classifiers are known as a "random forest" because several decision tree classifiers have been employed and each decision tree is modeled based on a random selection of features and training samples.

### Validation of the ML models

Both cross-validation during ML training and internal validation (test phase) after development of the ML models were done. Cross-validation is defined as dividing a dataset into k number of subsets. In one epoch, use k-1 subsets of data for training and use the remaining dataset to give an estimate of model skill while tuning model’s hyperparameters. For every epoch, validation dataset will be different, but it will be out of those *k* subsets of data. This is also referred to as *k*-fold cross-validation [13]. In this paper, fivefold cross-validation, was applied to the training set (4800 sample) and used to adjust the hyperparameters of each of the applied classifiers, where each fold consists of 960 samples.

Moreover, internal validation was done using the test dataset after the developments of the model. In internal validation, the test dataset is withheld from model training but is utilized to provide an unbiased evaluation of the quality of the final tuned model for comparing and selecting among different designed models.

Internal validation was tested on 1200 unseen samples using: accuracy and F1-score. Accuracy is the number of correctly predicted data points out of all the used test samples. F1-score is a way of combining the precision and recall of the model and is specially used to give a real evaluation for imbalanced data. It is defined as the harmonic mean of the model’s precision and recall, where precision refers to the fraction of correctly classified positive cases among all the estimated positive ones. Recall, also known as sensitivity, represents the fraction of samples classified as positive among the total number of positive examples [7].

## Results

### Performance comparison between single and ensemble models

In the 6000 observed HD sessions, complications occurred in 2874 sessions, while 3126 sessions were complication-free. The performance of various individual linear and non-linear algorithms was studied. 4800 samples of the data were used in the training process. The grid search technique, with fivefold cross-validation, was applied to the training set and used to adjust the hyperparameters of each of these classifiers. The grid search loops over a given range of values and measures the accuracy for each pair of values. It outputs the pair that gives the highest accuracy. The adjusted model was tested on 1200 samples, which were not seen by the model before, and the results are mentioned in this section. We compared the models according to the accuracy in the balanced dataset and F1-score in the imbalanced dataset. Furthermore, ensemble approaches with different subsets of data were applied to predict the occurrence of hemodialysis complications. The frequency of the 7 recorded complications is summarized in Additional file 1: Table S2.

Table 1 illustrates the performance of individual classifiers in predicting the occurrence of dialysis complications. The analysis showed that *SVC* and *MLP* with 97% accuracy have the best performance over the used classifiers without feature selection. However, it is too hard for nephrologists to collect 50 features per patient every session. Therefore, the performance of different sets of features was tested as the aim of this work is to use the smallest number of features that achieves the highest performance. The filter technique was used to choose the highest-ranking features.

**Table 1:** The performance of individual classifiers in predicting the occurrence of dialysis complications

Classifier		Accuracy	Precision	Recall	F1-score
MLP		97	97	97	97
KNN		94	94	94	94
SVC	Linear	97	97	97	97
	RBF	97	97	97	97
DT		95	95	95	95

**Fig. 3:**
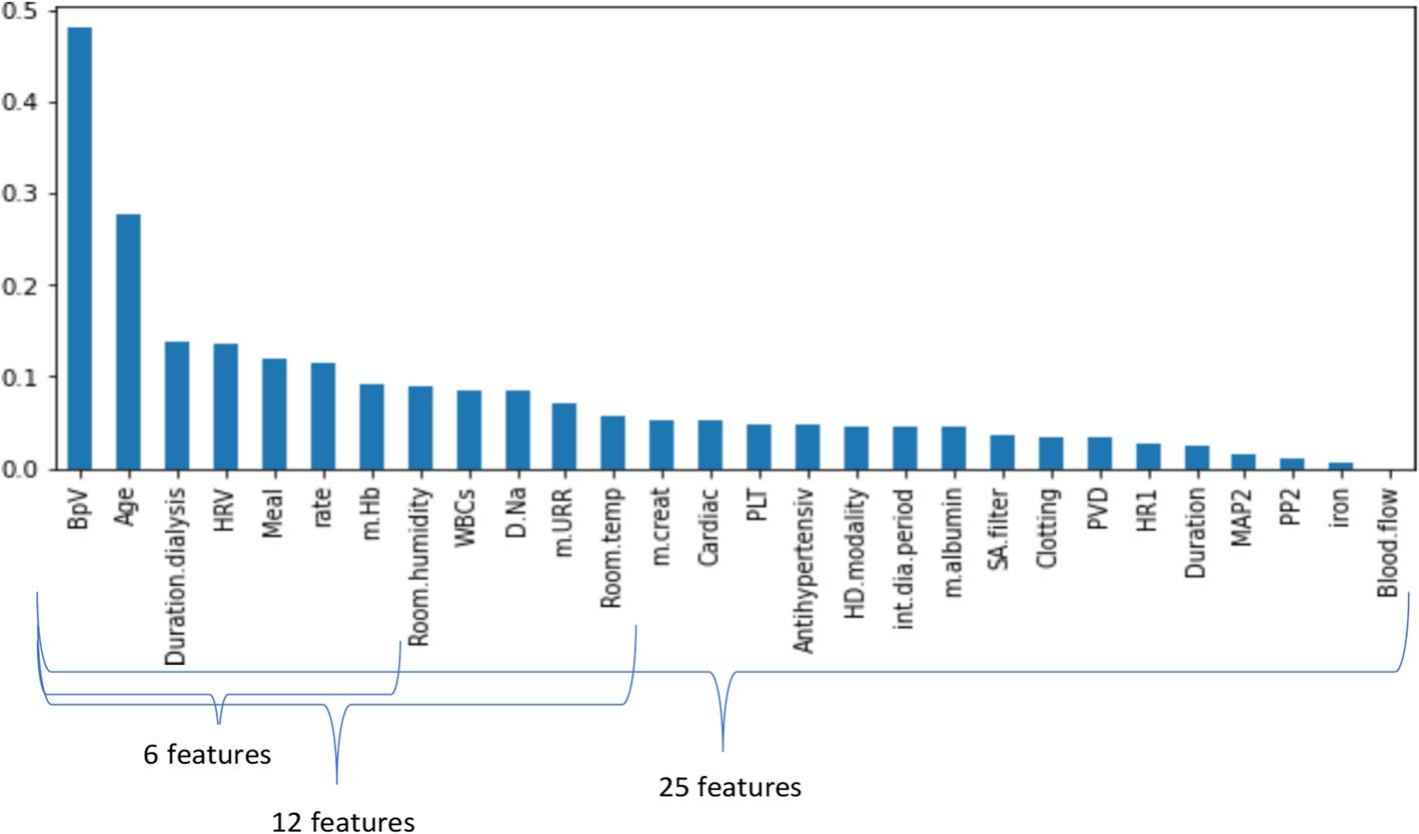
The most important studied features sorted according to rank

Figure 3 shows the highest-ranking 25 (half the number of the original features), 12, and 6 features that influence the occurrence of hemodialysis complications and sorts them according to their importance in the prediction model.

**Fig. 4:**
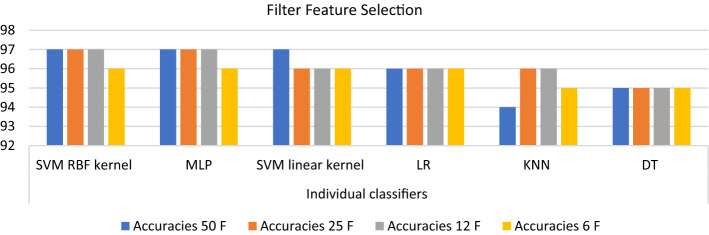
The accuracy of a different number of features using individual classifiers

Figure 4 presents the accuracy of using 50, 25, 12, and 6 features, respectively, with different individual classifiers. Accuracy is used to evaluate the performance of this dataset as it is the most common evaluation method to measure the performance of classifiers in the balanced dataset. The highest accuracy with 50, 25, 12, and 6 features are 97%, 97%, 97%, and 96%, respectively. While the total performance degraded with 6 features, using 12 features was the best subset in predicting the occurrence of complications without a significant compromise in accuracy as the accuracy of SVCR, MLP, DT was not reduced by using 12 features. In addition, the performance of KNN increased with the highly ranked 12 features. Reducing the number of features simplifies the model and allows its easy application.

**Fig. 5:**
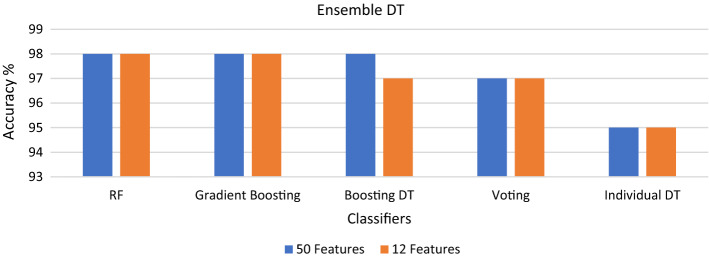
The accuracy of individual and ensemble decision trees

Figure 5 demonstrates the effect of the ensemble on the DT. Although DT alone was the worst classifier, when ensemble techniques were applied to the decision tree, they significantly improved its performance over other individual classifiers. The highest enhancement of 3% was achieved by the RF and GB, where accuracy rose to 98%, followed by voting with an increase of 2%. Furthermore, using 12 features, rather than 50, reduced the complexity of the model without degrading the accuracy of the DT, RF, GB, or voting ensemble, except when using boosting DT, where 12 features resulted in a 1% decline in accuracy.

Tables 2 and 3 list the F1-score for the occurrence of hypotension, hypertension, and dyspnea during the dialysis session using 50 and 12 features, respectively. F1-score was used as it is the most common evaluation method in the imbalanced dataset. GB had the highest F1-score in the 3 subset analyses.

**Table 2:** The F1-score of classifiers in predicting hypotension, hypertension, and dyspnea with **50** features

Complication	Learning model								
	SVCL	SVCR	KNN	DT	MLP	RF	GB	Boosting	Voting
Hypotension	87	91	84	82	90	89	92	89	88
Hypertension	86	86	83	72	90	88	94	88	89
Dyspnea	68	63	60	49	76	75	78	66	66

**Table 3:** The F1-score of classifiers in predicting hypotension, hypertension, and dyspnea with **12** features

Complication	Learning model								
	SVCL	SVCR	KNN	DT	MLP	RF	GB	Boosting	Voting
Hypotension	83	84	82	80	84	86	87	84	84
Hypertension	74	84	86	73	86	87	89	86	83
Dyspnea	49	49	56	49	55	54	67	56	53

### Training time

Tables 4 and 5 list the impact of using different models as well as 50 versus 12 features, on training time as a measure of model complexity. We calculated the training time, to determine the best model performance at the least training time.

**Table 4:** The training time in seconds of each classifier in the used datasets with **50** features

Complication	Learning model								
	SVCL	SVCR	KNN	DT	MLP	RF	GB	Boosting	Voting
Any complication (Hypotension, Headache, Hypertension, Cramps, Chest pain, Vomiting, or Dyspnea)	0.08	0.4	0.06	0.06	9.5	0.1	4	5	0.6
Hypotension	0.1	0.7	0.1	0.03	16	0.9	2.9	9	0.4
Hypertension	0.1	1.8	0.05	0.03	11.6	1.5	1.4	7.5	0.5
Dyspnea	0.1	0.2	0.05	0.02	10	0.5	1.6	4.5	0.4

**Table 5:** The training time in seconds of each classifier in the used datasets with **12** features

Complication	Learning model								
	SVCL	SVCR	KNN	DT	MLP	RF	GB	Boosting	Voting
Any complication (Hypotension, Headache, Hypertension, Cramps, Chest pain, Vomiting, or Dyspnea)	0.02	0.1	0.02	0.02	6.3	0.1	0.6	1.4	0.04
Hypotension	0.02	0.2	0.02	0.01	7	0.2	0.9	0.5	0.2
Hypertension	0.02	0.09	0.02	0.004	7	0.6	0.8	1.4	0.06
Dyspnea	0.03	0.1	0.02	0.01	6.9	0.4	0.7	1.2	0.06

The training time of all models trained with 50 features was greater than with 12 features, pointing out that a higher number of variables leads to an increase in training time and the model’s complexity as it leads to more complex mathematical computations. So, implementation of feature selection before training may significantly reduce training time and so computational power.

The time of GB using 12 features was 30%, 57%, and 44% less than the training time using 50 features in predicting hypotension, hypertension, and dyspnea, respectively. Furthermore, the training time of MLP is by far the highest in all algorithms. In the balanced dataset analysis, the shortest training time in the ensemble techniques, with the highest performance, was achieved by RF, while GB achieved the same accuracy but computationally took much more time. In the imbalanced dataset analyses, voting achieved the shortest training time, but with lower F1-score than GB and RF.

These differences in training time, with different number of predictors, different ML techniques and different datasets show how for most of the classifiers there is a trade-off between the classifier performance and the model computational complexity as indicated by training time.

## Discussion

In this study, five individual classifiers and three learners-based ensemble decision trees were compared in the prediction of hemodialysis complications using different subsets of data with 50, 25, 12, and 6 features. Twelve features were the least number that gave the best performance. As for training time, the time complexity when using 50 features was almost 4 times higher than 12 features in most models.

When a balanced dataset was used for the prediction of the occurrence of intra-dialytic complications, the priority was given to the accuracy, then the training time. The maximum accuracy achieved using single classifiers was 97% using MLP and SVC as represented in Fig. 4. However, the training time of MLP was extremely high as listed in Table 5. Therefore, SVC achieved the highest accuracy with minimum time complexity on the studied data.

The lowest accuracy was 95% obtained by DT. Therefore, an ensemble technique was applied to the DT to improve the accuracy. The accuracy was enhanced by 3% using the RF and GB reaching 98%, as shown in Fig. 5. However, the training time of RF was less than GB, as presented in Table 5. Therefore, the RF ensemble classifier outperforms the accuracy of a single classifier without increasing the complexity of the model.

In the imbalanced datasets, the prediction of hemodialysis hypotension, hypertension, and dyspnea, was assessed using the F1-score as well as the training time. The GB had the best F1-score in predicting hypotension, hypertension, and dyspnea as shown in Table 3. Furthermore, the multi-view characteristic of ensemble to the studied data resulted in enhancement in performance up to 12% as illustrated in Table 3, so for imbalanced case ensemble technique may be better for categorization.

In the case of ensemble models, GB had a longer training time than other ensembles such as RF but also gave a higher F1-score. The training time of GB with 12 features was less than with 50 features as represented in Table 5, but The F1-score with 50 features was greater than with 12 features. Therefore, there is a trade-off between the F1-score and the processing time when using a larger number of features.

Although ensemble approaches depend on multi-models decision, its complexity and training time may be better than some individual classifiers. Among all classifiers, MLP had the longest training time, which was over ten times larger than the training time of some ensemble classifiers, even with less features. This is due to huge numbers of weights and connection in MLP. Furthermore, to attain the highest performance, its architectures must be fine-tuned. In MLP, many design considerations must be taken, ranging from the number of layers to the number of nodes in each layer to the activation functions, and an architecture that works well for one issue does not always generalize well. In addition, the "black box" aspect of neural networks is definitely their most main shortcoming. Algorithms like DT, on the other hand, are quite interpretable. This is significant since interpretability is vital in the medical field with clinicians. So, enhancing the performance of this interpretable DT by applying ensemble is highly preferred in medical field.

Voting was in most scenarios faster than MLP and SVCR. Among the ensemble techniques studied, boosting computation was more complex and time consuming in comparison with bagging and voting. RF gave the shortest training time in balance datasets, voting gave the shortest time in imbalanced datasets, showing that the type of dataset may influence the choice of the most appropriate algorithm.

In comparison to the previously mentioned studies, our work has several strengths. Our work is the first to design a model for the prediction of intra-dialytic complications using this number of events. Recent studies with a larger number of sessions only tried to predict intra-dialytic hypotension [26, 33]. Instead of focusing just on the hypotension, our prediction model includes also several other intra-dialytic problems. Limited studies considered a multi-events problem [23, 24], however none reached the count of seven events as in our study.

In addition, most mentioned studies did not reach the same level of accuracy that our ensemble model reached in binary analysis.

Among the other strengths of our study is the consideration of environmental conditions as room temperature and humidity as well as meal consumption during dialysis, factors that are often overlooked and that proved significant. Temperature and humidity are especially important in the hot, humid climate of various developing countries.

Some of previous work [23, 24, 34] used advanced equipment such as non-contact sensor that are not commonly available in dialysis units so their model cannot be extensively implemented, unlike our model which relied on easily measured variables.

Most of the other studies examined fewer than the 50 features we explored and included lower numbers of studied sessions in comparison to the 6000 data records in our study. The larger dataset recorded, the more it is suitable for training. Use of multiple AI models also helped us reach the best accuracy with the least required features. Thus, our model after training can easily be implemented for complication detection in real-life sessions in a tenth of a second to identify the patients who need closer monitoring ultimately preventing these complications.

Although, this study demonstrates promising results, it has some limitations. This work was conducted in a single tertiary dialysis unit in Egypt, hindering its generalization. Further studies in other settings, whether regional or international, are necessary to externally validate and assure the generalizability of the suggested model. Despite the collection and consideration of 50 features, there may be multiple others that were not measured or assessed and that may affect outcome.

## Conclusion

Applying feature selection, different machine learning models and ensemble techniques decreased the number of features required and improved the accuracy of our model in the detection of intra-dialytic complications without increasing complexity. Random forest ensemble technique had the highest accuracy of 98% with the lowest training time using only 12 features. After external validation, the proposed model may help nephrologists to take appropriate action at a suitable time in patients to prevent its complications. Even though computer-aided techniques have improved in recent years, the choice of the ideal ML technique to each problem and dataset remains unclear. However, machine learning can be successfully used to analyze big medical data and allows the exploration of the effect of outcomes. Deep learning and ensemble techniques can be applied in multiple clinical scenarios to predict complications and aid in decision-making, eventually revolutionizing medicine.

## Supplementary Information

**Additional file 1:** **Table S1** Summary of the studied continuous variables. **Table S2** Summary of the studied categorical variables.

## Data Availability

Not applicable.

## Abbreviations

AdaBoost: Adaptive boosting
AI: Artificial intelligence
AKI: Acute kidney injury
ANN: Artificial neural network
AUC: Area under the curve
BP: Blood pressure
DT: Decision tree
ESKD: End-stage kidney disease
GB: Gradient boosting
HD: Hemodialysis
KNN: K-nearest neighbor
LR: Logistic regression
ML: Machine learning
MLP: Multi-layer perceptron
NB: Naïve Bayes
RAM: Random access memory
RF: Random forest
RRT: Renal replacement therapy
SBP: Systolic blood pressure
SVCL: Support Vector Classifier with Linear Kernel
SVCP: Support Vector Classifier with polynomial Kernel
SVCR: Radial basis kernel
SVM: Support vector machine
