# Supplementary material for: Early prediction of hemodialysis complications employing ensemble techniques
Source: Biomed Eng Online. 2022 Oct 11;21:74. doi: 10.1186/s12938-022-01044-0 (PMC9552449; doi:10.1186/s12938-022-01044-0)
Supplement: Supplementary file 1 — Additional file 1: Table S1 Summary of the studied continuous variables. Table S2 Summary of the studied categorical variables. [file 12938_2022_1044_MOESM1_ESM.docx]

**Table S1** Summary of the studied continuous variables

| **Features** | **Units** | **Mean** | **Standard deviation** |
| --- | --- | --- | --- |
| Heart rate 1st hour (HR1) | beat/minute | 81.2 | 12.7 |
| Heart rate 2nd hour (HR2) | beat/minute | 81.8 | 12.8 |
| Systolic blood pressure 1st hour (SBP1) | mmHg | 146 | 26.1 |
| Systolic blood pressure 2nd hour (SBP2) | mmHg | 142.4 | 26.4 |
| Diastolic blood pressure 1st hour (DBP1) | mmHg | 77 | 15.2 |
| Diastolic blood pressure 2nd hour (DBP2) | mmHg | 75.2 | 26.4 |
| Pulse Pressure 1st hour (PP1) | mmHg | 69 | 19.7 |
| Pulse Pressure 2nd hour (PP2) | mmHg | 75.2 | 15.9 |
| Mean amplitude pulse pressure 1st hour (MAP1) | mmHg | 100 | 17.2 |
| Mean amplitude pulse pressure 2nd hour (MAP2) | mmHg | 97.6 | 17.9 |
| Weight gain | Kg | 2.5 | 1.6 |
| Weight loss | Kg | 2.5 | 1.5 |
| Duration of session | Hour | 3.9 | 0.5 |
| Ultrafiltration rate (UFR) | L/h | 0.7 | 0.4 |
| Weight gain/ loss ratio | - | 0.7 | 0.5 |
| Dialysate sodium | mEq/L | 135.9 | 2.6 |
| Dialysate bicarbonate | mEq/L | 3.4 | 0.2 |
| Dialysate fluid temperature | 0C | 36.7 | 1.5 |
| Mean dialyzer blood flow | ml/min | 612.4 | 145.3 |
| Mean blood pump flow | ml/min | 337 | 49.8 |
| Hemoglobin | gm/dL | 9.9 | 1.9 |
| White blood cells (WBCs) | 109/L | 9 | 3.6 |
| Platelet cell count | 109/L | 258.2 | 92.6 |
| Urea reduction rate (m.URR) | % | 63.4 | 10.5 |
| Serum creatinine | mg/dL | 8.7 | 2.6 |
| Serum calcium | mg/dL | 8.6 | 1.4 |
| Serum phosphate | mg/dL | 4.5 | 1.5 |
| Serum albumin | g/dL | 3.2 | 0.7 |
| Room temperature | 0C | 24.6 | 4.6 |
| Room humidity | % | 47.5 | 9.4 |
| Age | Years | 50.5 | 16.4 |
| Duration since dialysis start | Years | 4.9 | 3.9 |

**Table S2** Summary of the studied categorical variables

| **Features** | **Value** | **Distribution (%)** |
| --- | --- | --- |
| Heart rate variability (HRV) | 0 (Stable) | 46.2 |
|  | 1 (Decreasing) | 23.6 |
|  | 2 (Increasing) | 30.2 |
| Blood pressure variability (BpV) | 0 (Stable) | 53 |
|  | 1 (Increasing) | 16.2 |
|  | 2 (Decreasing) | 30.8 |
| Peripheral vascular disease (PVD) | 0 (No) | 82.3 |
|  | 1 (Yes) | 17.7 |
| Heart disease | 0 (No) | 74.3 |
|  | 1 (Yes) | 25.7 |
| HD modality | 0 (Conventional Hemodialysis) | 69.9 |
|  | 1 (Hemodiafiltration) | 30.1 |
| Dialyzer membrane type | 0 (Low flux) | 51.5 |
|  | 1 (High flux) | 48.6 |
| Dialyzer membrane surface area (m2) | 0 (1.3) | 11.9 |
|  | 1 (1.5) | 23 |
|  | 2 (1.7) | 23.1 |
|  | 3 (1.8) | 23.3 |
|  | 4 (2.1) | 18.8 |
| Inter-dialytic period | 0 (1 day) | 62.2 |
|  | 1 (2 days) | 18.9 |
|  | 2 (3 days) | 14.1 |
|  | 3 (4 days) | 2.8 |
|  | 4 (6 days) | 2 |
| Clotting | 0 (No) | 88.1 |
|  | 1 (Yes) | 11.9 |
| Heparin | 0 (Free) | 8 |
|  | 1 (Initial) | 23 |
|  | 2 (Full) | 69 |
| Meal and drink | 0 (No) | 60.9 |
|  | 1 (Light) | 11.6 |
|  | 2 (Heavy) | 23.2 |
|  | 3 (Caffeine) | 4.3 |
| Antihypertensive medications | 0 (No) | 74.6 |
|  | 1 (Yes) | 25.4 |
| Epoetin alpha (dose/week-unites) (Medication used to treat anemia in ESRD patients) | 0 (No) | 16 |
|  | 1 (2000) | 17.7 |
|  | 2 (4000) | 45.4 |
|  | 3 (8000) | 13.4 |
|  | 4 (12000) | 7.5 |
| Iron medications | 0 (No) | 92.5 |
|  | 1 (Yes) | 7.5 |
| Diabetes | 0 (No) | 56.5 |
|  | 1 (Yes) | 43.5 |
| Original kidney disease | 0 (Hypertension) | 39.1 |
|  | 1 (Diabetes) | 28.6 |
|  | 2 (Glomerulonephritis) | 12.6 |
|  | 3 (Chronic kidney disease) | 8 |
|  | 4 (Drug) | 5 |
|  | 5 (Other) | 6.7 |
| Gender | 0 (Male) | 60.6 |
|  | 1 (Female) | 39.4 |
| Type of vascular access | 0 (Arteriovenous fistula) | 71.1 |
|  | 1 (Arterio-venous graft) | 19 |
|  | 2 (Permcath) | 1.5 |
|  | 3 (TempCath) | 8.4 |
| Complications **(Target)** | 0 (No complication) | 52.1 |
|  | 1 (Hypotension) | 16.4 |
|  | 2 (Headache) | 7.0 |
|  | 3 (Hypertension) | 7.4 |
|  | 4 (Cramps) | 5.8 |
|  | 5 (Chest pain) | 3.4 |
|  | 6 (Nausea/ Vomiting) | 4.6 |
|  | 7 (Dyspnea) | 3.3 |
